# Meet the Editorial Board Member

**DOI:** 10.2174/1570159X2204231106122340

**Published:** 2023-11-06

**Authors:** Gholamreza Azizi

**Affiliations:** 1 Alborz University of Medical Sciences, Karaj, Iran

   Gholamreza Azizi received his Ph.D. in biomedical sciences from the Tehran University of Medical Sciences. He is an investigator at the non-communicable diseases research center at Alborz University of Medical Sciences. His research focuses are on understanding the immunological mechanisms of immune regulation in patients with primary immunodeficiency and autoimmunity as well as the use of tyrosine kinases inhibitors to reduce inflammation in an animal model of multiple sclerosis.
